# Draft Genome Sequences of Antibiotic-Resistant Commensal *Escherichia coli*

**DOI:** 10.1128/genomeA.00873-14

**Published:** 2014-12-04

**Authors:** Meghan Garrett, Jennifer Parker, Craig M. Stephens

**Affiliations:** Biology Department, Santa Clara University, Santa Clara, California, USA

## Abstract

Antimicrobial resistance is a significant public health issue. We report here the draft genome sequences of three drug-resistant strains of commensal *Escherichia coli* isolated from a single healthy college student. Each strain has a distinct genome, but two of the three contain an identical large plasmid with multiple resistance genes.

## GENOME ANNOUNCEMENT

The antibiotic “resistome” within the normal human gut microbiome is thought to contribute to the spread of resistance genes to pathogens (1, 2). *Escherichia coli* readily moves between humans, animals, and the environment (3, 4), and its ability to propagate mobile genetic elements may make it a significant vector for the spread of resistance genes (5, 6). Here, we report the draft genome sequences for three *E. coli* strains isolated from an undergraduate student, as part of a microbiology course project. The strains were isolated from rectal swabs plated on MacConkey agar (Remel) with no antibiotic selection and then were screened for resistance to representatives of several classes of antibiotics using a disk diffusion assay (Hardy Diagnostics). *E. coli* strains CS02 and CS05, which were isolated months apart, are resistant to ampicillin, erythromycin, gentamicin, streptomycin, sulfamethoxazole, tetracycline, and trimethoprim. CS05 is additionally resistant to nalidixic acid. *E. coli* strain CS03, which was isolated from the same swab as CS02, is resistant to the β-lactams ampicillin, cephalothin, and the combination of amoxicillin and clavulanic acid.

Genomic DNA was isolated using the NucleoSpin tissue kit (Qiagen). Sequencing was performed on an Ion Torrent platform (Life Technologies) at the High-Throughput Genomics Center at the University of Washington. The average read length was ~140 bases. The read ends were trimmed in Geneious version 7.0.6 (Biomatters Ltd.) before *de novo* assembly by MIRA (version 4.0) (7), initially using 45% of the reads to facilitate rapid assembly. Contigs <500 bp were not included in the submission. Functional annotation was performed by the NCBI Prokaryotic Genomes Automatic Annotation Pipeline. The assembly metrics are provided in Table 1.

**TABLE 1:** Accession numbers and assembly metrics for annotated *E. coli* draft whole-genome sequences

Strain	NCBI accession no.	No. of contigs >500 bp	*N*_50_	No. of annotated genes	No. of predicted coding sequences
CS02	JNOF00000000	278	69,714	5,020	4,392
CS03	JNOG00000000	228	63,887	4,829	4,228
CS05	JNOI00000000	245	58,975	4,872	4,397

Resistance genes were identified in each strain by ResFinder version 2.1 (8). Two of the strains (CS02 and CS05) contain contigs nearly identical to most of *Klebsiella pneumoniae* plasmid pKF3-140 (9). Sequences in common with pKF3-140 include a 23-kb gene cluster encoding resistance to streptomycin-spectinomycin (*aadA5*), gentamicin [*aac(3)-Iind*], tetracycline (*tetA*), erythromycin (*mphA*), sulfonamides (*folP* alleles *sul1* and *sul2*), and trimethoprim (*dfrA17*). β-Lactam resistance attributable to *bla*_TEM-1B_ is chromosomally encoded. Non-plasmid-derived contigs from strains CS02 and CS05 share only 96 to 98% identity, and only strain CS05 is resistant to nalidixic acid, due to characteristic mutations in *gyrA*. Strains CS02 and CS05 were isolated at different times, and the genome sequences suggest that the plasmid responsible for multidrug resistance moved horizontally between distinct *E. coli* lineages. Further analysis of these strains will be presented in a future publication.

### Nucleotide sequence accession numbers.

This whole-genome shotgun (WGS) project has been deposited at DDBJ/EMBL/GenBank under the WGS accession numbers JNOF00000000 (CS02), JNOG00000000 (CS03), and JNOI00000000 (CS05). The versions described here are the first versions.
